# Fecal Transmission of Nucleopolyhedroviruses: A Neglected Route to Disease?

**DOI:** 10.3390/insects16060562

**Published:** 2025-05-26

**Authors:** Trevor Williams

**Affiliations:** Instituto de Ecología AC, Xalapa, Veracruz 91073, Mexico; trevor.williams@inecol.mx

**Keywords:** frass, *Baculoviridae*, *Alphabaculovirus*, midgut infection, Lepidoptera

## Abstract

Nucleopolyhedroviruses of lepidopteran larvae (*Alphabaculovirus*, *Baculoviridae*) form the basis for effective and highly selective biological insecticides for the control of caterpillar pests of greenhouse and field crops and forests. Horizontal transmission is usually achieved following the release of large quantities of viral occlusion bodies (OBs) from virus-killed insects. In the present review, I examine the evidence for productive midgut infection in different host species and the resulting transmission through the release of OBs in the feces (frass) of the host. This has been a neglected aspect of virus transmission since it was initially studied over six decades ago. The different host–virus pathosystems vary markedly in the quantity of OBs released in feces and in their ability to contaminate the host’s food plant. The release of fecal OBs tends to increase over time as the infection progresses. Although based on a small number of studies, the prevalence of transmission of fecal inoculum is comparable with that of recognized alternative routes for transmission and dissemination, such as cannibalism and interactions with predators and parasitoids. Finally, I outline a series of predictions that would affect the importance of OBs in feces as a source of inoculum in the environment and which could form the basis for future lines of research.

## Simple Summary

Lepidopteran nucleopolyhedroviruses are virulent pathogens of the larval stages of butterflies and moths, and they are widely used as the basis for biological insecticides. The virions are occluded within a matrix of protein to form highly resistant polyhedral occlusion bodies (OBs) that protect the virus outside of the insect. Larvae become infected after consuming foliage contaminated with OBs that dissolve in the intestine, release virions and infect gut cells, from where they spread to the cells of other tissues and cause a lethal disease. The virus-killed insect releases millions of progeny OBs into the environment for the following cycles of transmission. Here, I review the evidence that infected intestinal cells can produce biologically significant quantities of OBs that are released in the feces. These can be transmitted to other susceptible larvae and represent a little-recognized route for the transmission of these viruses. I compare fecal transmission with other alternative routes of transmission and make a series of suggestions for future lines of research to establish the importance of virus contamination of feces in the transmission and dispersal of these pathogens.

List of abbreviations, virus names and species names mentioned in this review.
AbbreviationCommon NameSpecies Name ^a^AcMNPVAutographa californica multiple nucleopolyhedrovirus*Alphabaculovirus aucalifornicae*AgMNPVAnticarsia gemmatalis multiple nucleopolyhedrovirus*Alphabaculovirus angemmatalis*BmNPVBombyx mori nucleopolyhedrovirus*Alphabaculovirus bomori*ChinNPVChrysodeixis includens nucleopolyhedrovirus*Alphabaculovirus alterchrincludentis*HearNPVHelicoverpa armigera nucleopolyhedrovirus*Alphabaculovirus helarmigerae*HypuNPVHyblaea puera nucleopolyhedrovirus*–*LdMNPVLymantria dispar multiple nucleopolyhedrovirus*Alphabaculovirus lydisparis*MbMNPVMamestra brassicae multiple nucleopolyhedrovirus*Alphabaculovirus mabrassicae*SeMNPVSpodoptera exigua multiple nucleopolyhedrovirus*Alphabaculovirus spexiguae*SfMNPVSpodoptera frugiperda multiple nucleopolyhedrovirus*Alphabaculovirus spofrugiperdae*TnSNPVTrichoplusia ni single nucleopolyhedrovirus*Alphabaculovirus trini*^a^ Species names are as defined by the International Committee on Taxonomy of Viruses [1]

## 1. Introduction

Lepidopteran nucleopolyhedroviruses (*Alphabaculovirus*; *Baculoviridae*) are virulent pathogens that are widely used as protein expression vectors [2] and as the basis for biological insecticides against lepidopteran pests of greenhouse and field crops and forests [3]. Viral nucleocapsids are enveloped to form occlusion-derived virions that are occluded within a crystalline matrix of polyhedrin protein to form occlusion bodies (OBs), typically 0.5–5 µm in diameter, that protect the virions in the environment for extended periods [4].

Horizontal transmission is achieved when larvae consume a lethal dose of OBs on plant foliage. The OBs dissolve in the alkaline midgut, cross the peritrophic matrix and infect midgut epithelial cells [5], from where they subsequently infect the cells of the trachea and most other tissues through the release of budded virions [4]. As the infection proceeds, OBs accumulate in the cell nuclei, and following death, millions of progeny OBs are released from the body of the insect for the following cycle of transmission [6].

The period between the initial infection and death varies according to the host–virus pathosystem, host instar, dose of OBs consumed, food plant and temperature [7] but typically lasts between 4 and 14 days. During the infection period, larvae continue to feed and move over the food plant in search of the most nutritious leaves [8,9]. During this phase, larvae can release viable virus in the feces, also known as “frass” [10]. As a result, plants on which infected larvae forage can harbor sufficient virus to initiate infections in conspecifics that inhabit the plant subsequently [11,12,13]. This echoes the importance of gut infection in sawflies infected by gammabaculoviruses or lepidopterans infected by cypoviruses that cause sustained defecation of virus-rich fecal matter, which is the primary source of disease transmission [14,15,16,17].

In contrast to the numerous studies on the acquisition of infection following spray applications of formulated OBs as biological insecticides, studies on the horizontal transmission of nucleopolyhedroviruses focus almost exclusively on the acquisition of infection by larvae that encounter OBs shortly after their release from virus-killed conspecifics [18,19,20,21,22]. This is because OBs from virus-killed larvae are the primary input of OB inoculum in the environment, representing many thousands of lethal doses concentrated over a small area of a plant. I will call this the “conventional” transmission cycle. In contrast, transmission from other sources of inoculum in the environment has received far less attention.

In the present review, I ask whether the release of viral OBs in feces is an ecologically relevant source of inoculum. For this, I review the evidence for, and processes involved in, OB production in feces and highlight the handful of quantitative studies. I then compare fecal OB production with established alternative routes for transmission and dissemination of OBs such as cannibalism and interactions with natural enemies. Finally, I identify a series of scenarios under which the fecal OBs are likely to influence virus ecology and the transmission of these pathogens in natural insect populations.

## 2. Evidence for OBs in Lepidopteran Feces

Insect bioassay has been the principal method applied to the detection of virus in feces. This involves feeding larvae with fecal samples collected from an infected donor larva, often at different intervals after the donor larva was inoculated with a lethal dose of OBs. The virus-induced mortality response in bioassay larvae is used as an indicator of the quantity of virus present in the fecal samples. Bioassay has two important advantages over alternative molecular or serological techniques. First, it is very sensitive for most host–virus pathosystems, as all but one of the studies reported to date have employed highly susceptible early instar larvae to detect virus in feces. Second, it only detects virus that is viable, i.e., that retains infectivity. Consequently, non-transmissible virus that has been inactivated during passage through the host gut will not register in bioassay results, unlike the results from molecular detection techniques based on the amplification of viral DNA [23] or the detection of viral proteins [24].

The presence of viral activity in the feces of nucleopolyhedrovirus-infected larvae was first detected in *Trichoplusia ni* [25]. Inoculation close to the 100% lethal concentration of TnSNPV OBs resulted in a 0–5% prevalence of lethal polyhedrosis in larvae that consumed feces sampled at 2–7 days post-inoculation, whereas a markedly higher inoculum resulted in 11–24% mortality in bioassay larvae (Table 1). This led Jaques [25] to conclude that contaminated feces were of little importance in the transmission of the virus, and this may have set the stage for the study of feces-mediated transmission as, over the six following decades, only five additional studies have attempted to detect viable virus in feces in other nucleopolyhedrovirus systems.

Twenty-five years later, a study on *Helicoverpa zea* and HearNPV (previously named HzSNPV) was performed in which low levels of virus-induced mortality (1.7–23.6%) were observed in bioassay larvae that consumed infected donor feces collected over a 4-day period (Table 1). Virus-induced mortality was even lower when the study was performed using leaf disks instead of an artificial diet [26].

By far the most cited article on this topic is that of Vasconcelos [11] who bioassayed the feces of MbMNPV-infected *M. brassicae* fourth instars sampled at 1–6 days post-inoculation and observed generally low levels of virus-induced mortality (0–11%). This study was, however, the first to adopt a rigorous statistical approach to quantifying insect responses to virus-contaminated feces.

Arakawa [27] later provided evidence that the feces of *Bombyx mori* contained significant quantities of BmNPV OBs on the day before death from polyhedrosis disease. Specifically, Arakawa [27] demonstrated that *B. mori* fourth instars that had been inoculated with a lethal concentration (LC_100_) of BmNPV produced feces containing approximately 1 × 10^5^ OBs/g. In this case, the sensitivity of the insect bioassay was increased by including polyoxin AL, a chitin synthesis inhibitor, to degrade the peritrophic matrix, thereby increasing the likelihood of infection of midgut cells by ODVs [28].

**Table 1:** Characteristics of studies on the presence of viral occlusion bodies (OBs) in feces in different host–virus systems.

Host/Virus	Larval Instar Used to Produce Feces (Instar Used to Bioassay Feces)	Day at Which Feces Sampled Post-Inoculation	Range of Virus- Induced Mortality Observed in Bioassay (%)	Estimated Quantity of OBs in Feces (OB/g)	Reference
*B. mori*/BmNPV	4th (3rd)	5	100 ^a^	~1 × 10^5 b^	[27]
*H. zea*/HearNPV ^c^	4th (1st)	1–4	1.7–23.6 (diet) 0.6–9.5 (leaf disk)	-	[26]
*H. puera*/HypuNPV	5th (5th)	0–2.5 ^d^	0–77	5 × 10^0^−2 × 10^7 e^	[29]
*M. brassicae*/MbMNPV	4th (2nd)	1–6	0–11 ^f^	-	[11]
*S. frugiperda*/SfMNPV	4th (2nd)	2–6	3.9–68.3	5.4 × 10^3^−4.4 × 10^6^	[30]
*T. ni*/TnSNPV	3rd (3rd)	1–5	11–25 ^g^	-	[25]

^a^ Mortality observed in 10^−1^ dilution of feces. ^b^ Estimated from data in Table 4 in Arakawa [27] and Table 1 in Arakawa and Nozawa [28]. ^c^ HzSNPV was renamed HearNPV, a recognized species in the *Alphabaculovirus* genus [31]. ^d^ Samples taken at 6 h intervals up to 60 h post-inoculation (2.5 days). ^e^ OBs/mL in water, direct counts on samples were performed in a hemocytometer. ^f^ Values estimated from figure in Vasconcelos [11]. ^g^ Values from larvae inoculated with 5 × 10^6^ OBs. Lower doses of inoculum resulted in just 0–5% mortality in bioassay larvae that consumed feces sampled at 2–7 days post-inoculation.

This was followed by the study by Bindu et al. [29] in which OBs of HypuNPV were counted directly from a centrifuged preparation of larval feces. This study was unusual in that high concentrations of OBs were observed in fecal samples, reaching up to 2 × 10^7^ OBs/mL for fecal samples suspended in water at 2.5 days post-inoculation (values estimated from figure in Bindu et al. [29]). This resulted in up to 77% mortality of larvae that consumed fecal samples (Table 1).

The most recent study is that of Avila-Hernández et al. [30] who detected up to 4.4 × 10^6^ OBs/g of SfMNPV in feces of diet-fed *Spodoptera frugiperda* larvae with up to 68% mortality in the larval bioassay (Table 1). An alternative quantification technique based on quantitative PCR gave estimates of between 1.5 × 10^3^ and 5.3 × 10^5^ OBs/g of feces, which was about 10-fold lower than the bioassay-derived estimates, likely due to a combination of inhibition of the amplification reaction by fecal contaminants and loss of viral DNA during sample processing.

**Figure 1:**
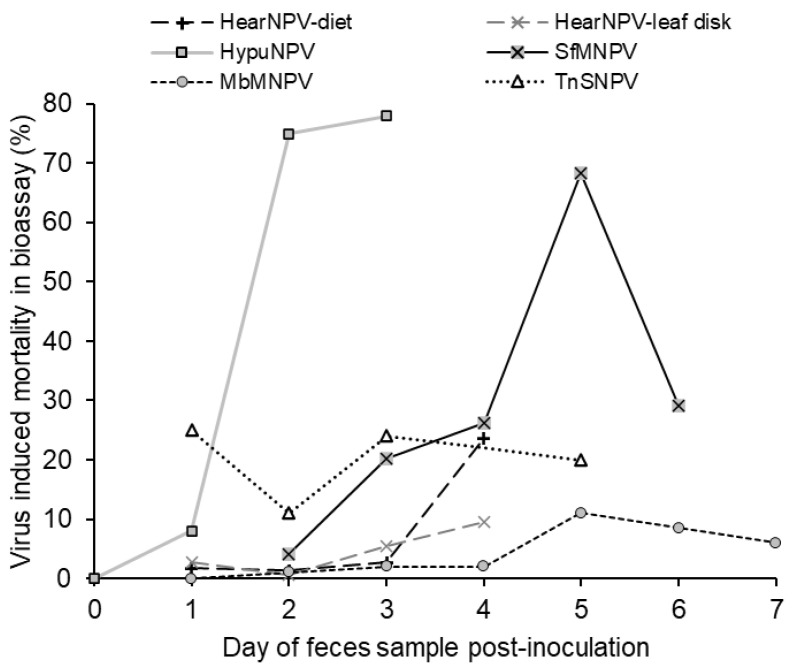
Virus-induced mortality observed in bioassays of fecal samples collected at different times post-inoculation. The details of each study are provided in Table 1.

All but one of the studies to date have noted that the presence of viable virus in feces increases during the larval infection period (Figure 1). Usually, a gradual increase is observed in the mortality of larvae that consume feces samples taken over the course of several days, reaching a peak shortly before death of the infected donor insect. This presumably reflects the progress of disease in the host gut.

Several studies have reported the detection of low levels of viable virus in feces collected as soon as 24–48 h post-inoculation [26,30], whereas others only detect viable virus later in infection [11] or immediately prior to death [27]. In this respect, the study on HypuNPV in *H. puera* is unusual in that the virus in feces rapidly increased between 24 and 60 h post-inoculation (plotted as 3 days in Figure 1) [29], although this is a highly virulent virus with an unusually rapid speed of kill of 72–84 h in third instars [32]. Consequently, the study on HypuNPV contrasts with the findings on the other host–virus systems and may be considered atypical. In the early study by Jaques [25], a carry-over of virus activity from the TnSNPV inoculum was detected at 1 day post-inoculation followed by a variable prevalence of mortality (11–24%) in the insect bioassay in samples taken over the following 5 days at the highest inoculum tested (Figure 1).

Importantly, most studies on the detection of nucleopolyhedrovirus in feces have involved surface decontamination of the host larvae by brief immersion in sodium carbonate or sodium hypochlorite solution to avoid carry-over of OBs from the original inoculum. In the absence of surface decontamination, virus activity in feces collected shortly after inoculation (<24 h) has been attributed to the presence of residual inoculum that contaminated the larval body or remained viable after passage through the gut [11,25,26].

## 3. How Does Fecal Inoculum Compare to “Conventional” Transmission?

So how does the quantity of OBs released in feces compare to the quantity of OBs released following death of the infected insect? Only two studies provide information on the total production of fecal OBs over the course of a nucleopolyhedrovirus infection. This information was obtained from the data on HypuNPV (Supplemental Table S1; [29]) and the values for feces production and OB concentration in feces reported for SfMNPV (Supplemental Table S2; [30]).

**Figure 2:**
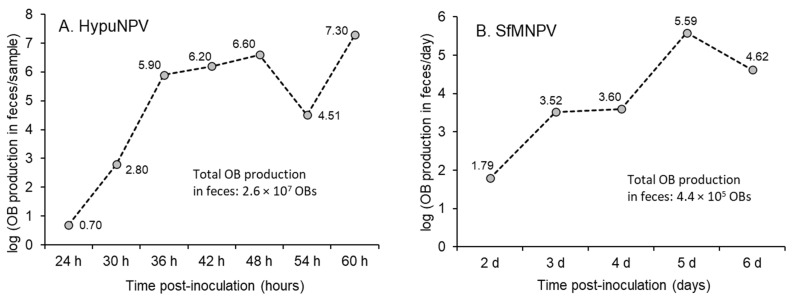
Logarithm of the quantities of OBs produced in feces at each sample time point for (**A**) HypuNPV and (**B**) SfMNPV in their homologous hosts. The total OB production in feces was obtained by adding the estimated production at each time point over the course of the infection prior to death (Supplemental Tables S1 and S2).

Using this approach, the total OB production in the feces of *H. puera* fifth instar larvae was estimated at 2.6 × 10^7^ OBs (data summed over all time points in Figure 2A), which compares to 7.8 × 10^8^–1.5 × 10^9^ OBs produced in a virus-killed fifth instar larva [32], a difference of 30–57-fold. Following the same approach, the total fecal OB production in *S. frugiperda* fourth instar larvae was estimated at 4.4 × 10^5^ OBs (data summed over all time points in Figure 2B), which compares to 1.0 × 10^9^ OBs produced in a virus-killed fourth instar larva [33], a difference of approximately 2200-fold.

## 4. Are the Quantities of Virus in Feces Biologically Significant?

Given the markedly lower abundance of OBs in feces compared to a virus-killed insect of the same instar, the question arises as to whether the OBs released in feces represent a biologically significant quantity of inoculum for transmission of these pathogens. The evidence comes from experiments performed on insect diet and the insect’s food plants.

In laboratory tests on artificial diet, the stage of the healthy test larvae was matched to that of each infected larva [34]. Each infected larva also underwent surface decontamination with hypochlorite treatment to avoid carry-over of inoculum. Cohabitation of healthy and AgMNPV-infected larvae in cups containing diet resulted in 5.8–9.3% acquisition of lethal infection in *A. gemmatalis* irrespective of the larval instar involved. In contrast, cohabitation by larvae of *Chrysodeixis includens* resulted in 30.5% acquisition of ChinNPV infection in first instars but was markedly lower (0–3.9%) in tests involving later instars. In these tests, the infected larva was removed at 24 h prior to death, so the main route of transmission was likely to be contaminated feces produced by the single infected larva in the group. Similarly, larvae of *M. brassicae* that consumed the diet in cups that an infected conspecific had previously inhabited for 24 h acquired 7%, 9% and 44% infection when the infected larvae were at 4, 5 and 6 days post-inoculation, respectively [11]. In this case, obvious fecal material was removed prior to the test but the diet surface was clearly contaminated, nonetheless.

Of the *Spodoptera exigua* larvae that foraged on sweet pepper plants that had previously been inhabited by an SeMNPV-infected conspecific larva, 50% of larvae acquired lethal polyhedrosis disease despite having no contact with the virus-killed insect. This was attributed to foliage contamination from the feces and regurgitated residues of the original infected larva [12]. Similarly, 22% of *A. gemmatalis* third instars acquired a lethal infection after foraging for 24 h on soya plants that had previously been inhabited by an infected conspecific larva [13]. The acquisition of MbMNPV infection in *M. brassicae* second instars increased from 0.9 to 6.5% when larvae foraged over plants that had been infested by an infected fourth instar conspecific in the period 1–3 days post-inoculation or 1–6 days post-inoculation, respectively, indicating that the quantity of virus released later in infection had significantly increased in this system [11]. Likewise, the acquisition of infection in *S. frugiperda* second instars increased from 2.5 to 48% after larvae foraged on maize leaf sections that were previously inhabited by infected fourth instars at 3 and 4 days post-inoculation, respectively [30].

These studies mention the possible release of the virus in the saliva or regurgitated material of infected larvae as a potential source of inoculum. Regurgitation is a recognized antipredator behavior in Lepidoptera [35], but the only study to have attempted to detect nucleopolyhedrovirus in saliva and regurgitated material concluded that only very small amounts of virus were present in the regurgitated material of *T. ni* third instars that had been inoculated with TnSNPV at 1–4 days previously [25]. Similarly, no evidence of AcMNPV replication in the salivary glands was observed in infected *T. ni* larvae up to 60 h post-inoculation [36].

It is clear then that the release of virus-contaminated feces is sufficient to initiate lethal disease in susceptible conspecific insects that feed on these food plants. Moreover, heterogeneity in the spatial distribution of inoculum affects the likelihood of transmission, with uniformly distributed inoculum resulting in more transmission than highly aggregated inoculum [37]. Consequently, although each virus-killed insect represents an abundant but highly localized inoculum, the release of lower quantities of OBs in fecal material distributed over the food plant reflects the movement of the infected insect during its development and feeding, which should increase the likelihood of healthy larvae consuming contaminated foliage and favor the transmission of fecally derived inoculum.

## 5. How Does Virus Activity Appear in Feces?

How does virus appear in the fecal material and how does this change over the course of nucleopolyhedrovirus infection? The proliferation of infection in the midgut varies across host–virus systems. Early studies indicated that midgut cells were never or rarely involved in the production of OBs in some hosts [38,39], or midgut production of OBs was only transient [36,40], whereas in other species, such as *A. gemmatalis*, midgut replication is abundant [41].

Infection foci begin as single infected cells which transmit the infection to the neighboring cells via a GP64-mediated process so that by 48 h post-inoculation, distinct foci are present [42]. This appears to be concurrent with the processes that target the establishment of systemic infection in host tracheal cells [43]. The extent of midgut infection is dose-dependent with single or few foci of infection at low doses of OBs compared to extensive infection following ingestion of high doses of inoculum [42]. In contrast, ingestion of intermediate doses resulted in high levels of infection in the anterior midgut in over 70% of *T. ni* larvae with markedly fewer larvae (<20%) developing extensive infection in the posterior section of the midgut [42]. In a different study, *S. exigua* third instars inoculated with 10^6^ OBs/mL of recombinant bacmid OBs had approximately 10–40 obvious foci of infection in the midgut at 72 h post-inoculation [44].

The spatial distribution of infection likely reflects a combination of factors including the distribution of susceptible columnar cells, the regionalization of different cell types and variation in the abundance of ODVs that traverse the peritrophic matrix along the midgut [42]. Single-nucleus RNA sequencing techniques have now revealed that all cell types present in the *B. mori* midgut are infected at 72 h post-inoculation but with clearly higher viral loads in columnar cells compared to goblet cells and other cell types [45].

The host response involves sloughing and replacing infected cells as a means of limiting or voiding the infection [46]. This response begins as soon as 16 h post-inoculation in some hosts [47] but increases as the infection progresses [42]. Consequently, the principal source of viral activity in the feces of infected larvae is due to the sloughing and lysis of cells expelled from infection foci. Larvae also empty the midgut lumen and expel sloughed infected cells immediately before each molt [47], so that studies on fecal OBs may expect an increase in fecal OBs prior to molting.

**Figure 3:**
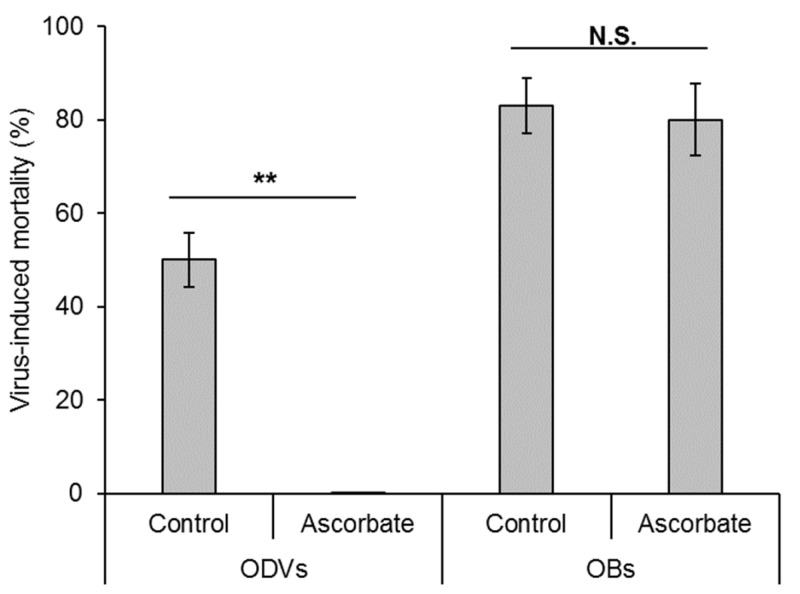
Sodium ascorbate treatment of ODVs resulted in complete loss of activity, whereas treatment of OBs had no significant effect on mortality of *Bombyx mori* third instars in a laboratory bioassay. Error bars indicate SE. Data on ODVs from experiment 1 (Table 2, 10^0^ dilution) and data on OBs from experiment 4 (Table 3, 10^−2^ dilution) in Arakawa [27] (Welch’s *t*-test, N.S. *p* > 0.05, ** *p* = 0.01).

It has been argued that practically all the viral activity in the feces of larvae exists in the form of OBs rather than free ODVs or even budded virions [30]. Support for this comes from the direct counting of OBs in feces [29] and from an experimental approach by Arakawa [27] in which ODVs were completely inactivated by treatment with sodium ascorbate, a strong reducing agent that generates hydroxyl radicals during autoxidation; OBs, however, were not affected by ascorbate treatment (Figure 3). Using this technique, Arakawa [27] demonstrated that the viral activity in *B. mori* feces collected at 4 h post-inoculation was due to the presence of BmNPV ODVs released from the original inoculum, whereas the activity present in feces collected at 5 days post-inoculation was due to OBs. The deactivation of budded virions and ODVs by ascorbic acid and glutathione was also shown to be effective in AcMNPV and TnSNPV, whereas treated OBs fully retained their activity [48]. To date, however, this potentially useful technique has found no uptake in the baculovirus research community.

To a large degree, studies on lepidopteran feces agree that the passage of viral OBs through the insect gut eliminates most of the original activity present in the inoculum. This is likely due to a combination of dilution of virus particles in the gut contents and the harsh environment of the gut that employs an array of antimicrobial defenses. So, the question arises, how do OBs released from infected midgut cells remain viable during gut transit and defecation?

The antiviral measures in the midgut include the alkaline pH generated by goblet cells in combination with a carbonic anhydrase that generates a strong bicarbonate ion flux [49,50]. This alkalization is strongest in the anterior and central regions of the midgut and weakest in the posterior region immediately prior to the hindgut [51,52]. An array of enzymes with antiviral activity are also secreted into the anterior midgut including potent serine proteases [53,54], as well as amylases, lysozymes, lipases and endo- and exopeptidases [55,56] and additional enzymes in the central and posterior sections of the midgut [57,58].

Given these circumstances, it would appear that OBs may find a refuge from adverse conditions if they are mainly released from cells in the posterior midgut, or if the sloughed cell itself offers temporary protection from enzymatic degradation, or if the OBs remain close to the epithelium as they travel along the midgut as the peri-epithelial space has a pH 2.5–3 units closer to neutral than the material within the peritrophic matrix [59]. In addition, infection by HearNPV and AcMNPV both result in marked downregulation of transcription in the gut of their hosts including genes for digestive enzymes [53,60], so the infected gut may be a less hostile environment to OBs than it otherwise would be. This is an issue that merits closer examination.

## 6. Importance of Fecal Contamination Compared to Other Alternative Transmission Routes

I would argue that fecal OBs are just as important as other alternative routes of transmission and dispersal that have been studied in some detail. For example, the frequency of cannibalistic behavior resulting in virus transmission ranged from just 3.3% in *M. brassicae* [11] to ~30% in *S. frugiperda* [61,62] and a range of prevalences in highly cannibalistic species such as *H. armigera* [63] and *H. zea* [64], depending on the experimental conditions.

Insect predators have also been demonstrated to be capable of dispersal of OBs after feeding on virus-infected larvae. This is because the transit of OBs through the acidic gut of most predatory insects does not adversely affect the activity of OBs. The quantities of OBs in the predator’s feces are similar to the range of those reported for the feces of infected lepidopteran hosts (see Figure 2) with 5 × 10^4^–1 × 10^8^ OBs of AgMNPV in the feces of pentatomid bugs [65], 5.3 × 10^6^ OBs in the feces of a nabid bug [66] and 5 × 10^3^–2.2 × 10^7^ OBs released in the feces of carabid beetles that fed on AgMNPV-infected prey [67].

The OB-contaminated feces of these predators are also sufficient to initiate infections in host larvae that forage on the same plants as the predators. For example, the prevalence of lethal polyhedrosis disease transmitted to larvae varies widely, from 4.7% in *S. frugiperda* [68] to 2.8–6.5% in *M. brassicae* [69], 6.7–16.8% in *A. gemmatalis* [66], 13% in *Spodoptera litura* [70], 14% in *T. ni* [71] and 20–63% or more in *S. exigua* [72], depending largely on the predator, the experimental conditions and the interval between consumption of the virus-infected prey and defecation.

In a similar vein, parasitoid-mediated transmission of nucleopolyhedroviruses has attracted considerable interest in its potential contribution to biological pest control. Parasitoid wasps that oviposit in infected hosts can transmit the infection to several or many of the larvae that they probe or parasitize subsequently [73]. The prevalence of transmission of infection tends to decrease as the interval between the initial contamination of the ovipositor and subsequent stinging events increases. A moderate prevalence (10–60%) of such transmission has been reported in ichneumonid [74,75] and braconid parasitoids [76,77,78,79], compared to a lower prevalence in a eulophid [77]. Parasitoids that manage to complete their development in virus-infected hosts can also transmit the infection to susceptible hosts at high prevalence (>60%) [74,80].

On occasion, it has been argued that predator- and parasitoid-mediated dissemination of nucleopolyhedroviruses contributes little to the overall control of pests [81]. However, field studies following the introduction of nucleopolyhedrovirus to a particular crop have revealed that the spread of these viruses is correlated with the movement of predatory arthropods and parasitoids, suggesting that natural enemies significantly influence the rate and distance of viral dissemination [82,83]. The rate of dispersal has also been quantified for virus-contaminated predators and scavenging flies in microcosm experiments [84]. In addition, a high prevalence of contamination of predators over periods lasting several weeks in the soybean–*A. gemmatalis* system [85] contrasts with a low prevalence of contamination and little predator movement in soybean infested by *Helicoverpa*/*Heliothis* spp. [81], suggesting that system-specific factors may modulate the importance of natural enemy–nucleopolyhedrovirus interactions. Nonetheless, accurate determination of the relative contribution of natural enemy dispersal and fecal OBs to the transmission and spread of infection clearly requires quantitative studies across a range of host, pathogen and natural enemy densities.

## 7. Future Lines of Research

Given the available information and an understanding of nucleopolyhedrovirus biology and ecology, it is possible to envisage a series of scenarios or predictions that would influence the importance of fecally mediated transmission of these viruses. I outline each of these as proposals for future lines of research.

### 7.1. Virus Activity in Feces Reflects Midgut Replication

From the limited number of studies available, it appears that host–virus systems may be divided into those with sparse or transient midgut replication such as *T. ni* [36,38] compared to others with abundant reproduction such as *A. gemmatalis* [41]. Midgut replication appears to be correlated with viral activity in the feces (Figure 2) or the ability to contaminate food plants in the case of SeMNPV or AgMNPV [12,13], although the paucity of studies makes firm statements impossible.

### 7.2. Feeding Behavior Will Affect the Transmission of Fecal OBs

Aggregated feeding habits mean that nucleopolyhedrovirus in feces is more likely to be transmitted in gregarious species such as those in the families Nymphalidae, Pieridae, Papilionidae and Lasiocampidae (tent caterpillars) [86] compared to solitary-feeding species. However, Hochberg [87] has argued that gregarious feeding species have evolved higher resistance to their viruses than solitary species precisely because the risk of transmission within the feeding group is elevated. For solitary species, fecal OBs are more likely to be transmitted at high local population densities when several larvae feed on a shared host plant.

### 7.3. Plant Architecture and Larval Feeding Habits Will Determine the Accumulation of Fecal OBs

**Figure 4:**
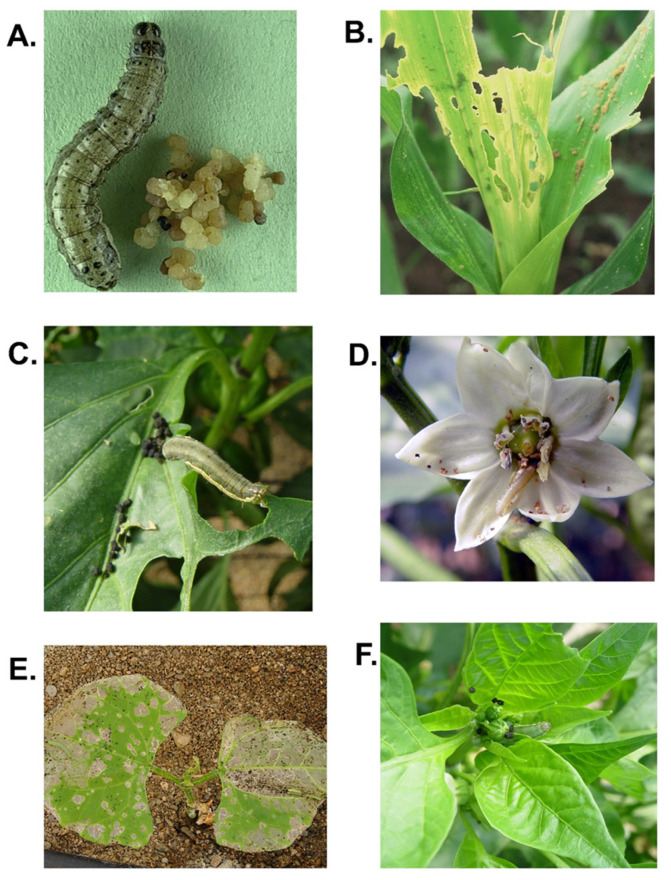
Lepidopteran feces produced when feeding on an artificial diet or plants. (**A**) Feces produced by *Spodoptera frugiperda* larva on an artificial diet. (**B**) Feces of *S. frugiperda* contaminate the leaf whorl of a maize plant. Feces of *S. exigua* larvae accumulate in the (**C**) leaf axil and (**D**) flower of a sweet pepper plant and (**E**) over the surface of leaves of a cucumber plant and (**F**) around the buds of sweet pepper plants.

The spatial distribution of OB-contaminated feces will reflect larval feeding habits. Although virus-contaminated feces may fall to the ground and contribute to the soil reservoir of OBs [88], plant architecture may favor the accumulation of fecal material in leaf axils, buds, flowers and leaf whorls or deeply grooved leaves (Figure 4A–F). These sites are preferred by lepidopteran larvae due to their nutritive characteristics [89,90] and may also harbor increased concentrations of fecal OBs. A similar argument could apply to viruses infecting stored product pests in which the feces are often expelled from grains or seeds as dust that remains in the dry protected environment of stored products for extended periods. This idea finds support from an electron microscope study in which granulovirus (*Betabaculovirus*) particles were shed into the midgut lumen from infected epithelial cells late in infection and presumably defecated by *Plodia interpunctella* larvae [91].

### 7.4. Physical and Chemical Properties of Feces May Affect OB Persistence

OBs in fecal residues may benefit if the fecal material protects OBs from solar ultraviolet radiation [92] or adverse phylloplane chemistry [93,94], both of which can rapidly inactivate unprotected OBs. Indeed, lepidopteran feces can be rich in flavonoids [95] that have ultraviolet protective properties [96]. In addition, both the opaque nature of lepidopteran feces and the physical separation from the leaf surface that feces offer to OBs could favor OB persistence on plant surfaces and thereby increase their probability of transmission.

### 7.5. Conspecific Attraction to Fecal Material

OBs in feces may be consumed preferentially if the feces themselves are attractive to conspecific larvae. Feces are often retained in the nests of webworms, tent caterpillars and some pyralids, whereas others make considerable efforts to cast the fecal pellets away from feeding areas in an attempt to avoid attracting natural enemies [97]. However, several species are attracted to conspecific larval feces, including *Spodoptera littoralis* [98]. Bacteria in fecal matter produce several volatile compounds including 2-methoxyphenol (guaiacol), which is highly attractive to *S. littoralis* larvae and could promote coprophagy and virus acquisition in conspecific insects. In addition, the immune response of nucleopolyhedrovirus-infected larvae is suppressed, resulting in increased bacterial populations [99] and possibly an increased production of attractive volatile compounds in the feces of infected larvae, which would also promote coprophagous transmission of the virus. The responses of lepidopteran larvae to the remains of virus-killed conspecifics have been examined in several species [12,21,100], but responses to OB-contaminated feces remain largely unstudied.

## 8. Conclusions

Although few in number, all the studies to date report the presence of OBs in lepidopteran feces, the quantities of which vary markedly across host–virus pathosystems. Defecation by infected larvae prior to their death can result in an appreciable prevalence of mortality in larvae that inhabit the plant subsequently. As larvae roam across plants in search of nutritious leaves, OBs in feces may be spread across different feeding sites and may be concentrated by some aspects of plant architecture to increase their probability of transmission. I conclude that virus transmission prior to death of the primary infected insect is a neglected but potentially intriguing and biologically relevant area of research.

## Data Availability

No new data were created or analyzed in this study. Data sharing is not applicable to this article.

## Supplementary Materials

The following supporting information can be downloaded at: https://www.mdpi.com/article/10.3390/insects16060562/s1, Table S1: Quantities of HypuNPV OBs counted in fecal samples of *Hyblaea puera* fifth instar larvae at 24–60 h post-inoculation; Table S2: Quantities of SfMNPV OBs estimated in fecal samples of *Spodoptera frugiperda* fourth instar larvae at 2–6 days post-inoculation.
