# Supplementary material for: Fecal Transmission of Nucleopolyhedroviruses: A Neglected Route to Disease?
Source: Insects. 2025 May 26;16(6):562. doi: 10.3390/insects16060562 (PMC12192623; doi:10.3390/insects16060562)
Supplement: Supplementary file 1 [file insects-16-00562-s001.zip › insects-3635988-supplementary.pdf]

Supplemental Table S1: Quantities of HypuNPV OBs counted in fecal samples of *Hyblaea puera* fifth instar larvae at 24 – 60 h post-inoculation. Data estimated from figure 1 in Bindu et al. [29].

	Sample time (hours)							Total OB production
	24 h	30 h	36 h	42 h	48 h	54 h	60 h	
Number of OBs produced in feces at each time point	5×10^{0}	6.3×10^{1}	7.9×10^{5}	1.6×10^{6}	4.0×10^{6}	3.2×10^{4}	2.0×10^{7}	2.64×10^{7}
Log (OB production)	0.6989	2.7993	5.8976	6.2041	6.6020	4.5051	7.3010	

Supplemental Table S2: Quantities of SfMNPV OBs estimated in fecal samples of *Spodoptera frugiperda* fourth instar larvae at 2 – 6 days post-inoculation. Data estimated from values in Avila-Hernández et al. [30].

	Sample time (days)					Total OB production
	2 d	3 d	4 d	5 d	6 d	
Number of OBs present in 100 mg feces; estimated by bioassay	5.4×10^{2}	1.2×10^{4}	2.1×10^{4}	4.4×10^{5}	2.6×10^{4}	
Mean quantity of feces produced per larva per day (mg)	11.5	27.8	19.1	87.7	161.4	
Number of OBs produced in feces at each time point	6.2×10^{1}	3.3×10^{3}	4.0×10^{3}	3.9×10^{5}	4.2×10^{4}	4.35×10^{5}
Log (OB production)	1.7931	3.5232	3.6032	5.5865	4.6229	
